# Optimization of Volatile Compounds Extraction from Industrial Celery (*Apium graveolens*) By-Products by Using Response Surface Methodology and Study of Their Potential as Antioxidant Sources

**DOI:** 10.3390/foods10112664

**Published:** 2021-11-02

**Authors:** Ana Beltrán Sanahuja, Mercedes Ponce Landete, María Isabel Domingo Martínez, María Soledad Prats Moya, Arantzazu Valdés García

**Affiliations:** Analytical Chemistry, Nutrition and Food Sciences Department, University of Alicante, P.O. Box 99, E-03080 Alicante, Spain; ana.beltran@ua.es (A.B.S.); mpl68@gcloud.ua.es (M.P.L.); midm6@gcloud.ua.es (M.I.D.M.); maria.prats@ua.es (M.S.P.M.)

**Keywords:** HS-SPME, Box–Behnken, volatile compounds, antioxidant activity, phenolic content, ABTS, FRAP, celery root and stalk, agricultural by-products

## Abstract

In this study, the potential of industrial celery by-products (the stalk and root) serving as sources of aromatics and antioxidants was investigated. A headspace solid phase microextraction–gas chromatography–mass spectrometry (HS-SPME–GC–MS) procedure was optimized to isolate volatile compounds from celery by-products. A Box–Behnken experimental design was proposed to optimize the procedure through a response surface methodology. The optimal extraction conditions were found to be 1.6 g of homogenized fresh by-product at 30 °C for 60 min. Under these conditions, 26 volatile compounds in stalk and root samples were identified, monoterpenes and sesquiterpenes being the main components. The content of limonene and γ-terpinene found in the stalk was significantly higher in comparison with root samples. Total phenolic content and antioxidant activity (ABTS and FRAP) results underlined the celery wastes studied as good sources of free radical scavengers. This work suggests the potential application of these by-products in the food industry and opens new pathways to valorize celery residues, contributing to the circular economy.

## 1. Introduction

Annually, almost 1.3 billion tons of food, a third of worldwide food production, is not used to be eaten. Fruits and vegetables accounted for up to 45% of the whole foods. Such wastage is becoming a serious economic and environmental problem [1]. Thus, agricultural activities require the development of new valorization processes of food industrial residues in order to contribute to food industry sustainability and the circular economy system [2].

The most prominent of Apium plants (from the family *Apiaceae*) is the *Apium graveolens* L., popularly known as celery [3]. It is an annual or biennial herb species that grows in Europe, Africa and Asia, and is consumed worldwide [4,5], most frequently as a raw material in salads or in cooking as a condiment [6]. Celery is meanly formed of water (95%) and dietary fiber and it is also rich in antioxidants [7]. Different studies have been focused on evaluating the volatile composition of different celery cultivars [8,9] and parts of the plant, such as leaves, stalk and seeds [10,11,12], since this combination has been associated with its particular flavor. A review reported by Turner et al. [6] revealed that terpenes, phthalides, alcohols, esters and aldehydes are responsible for the fresh, grassy and green notes of the celery plant. However, the study of the volatile composition of stalk and root industrial wastes has not been reported in the literature. In this work, we propose the optimization of the volatile extraction step using a headspace solid phase microextraction (HS-SPME) coupled with gas chromatography–mass spectrometry (GC–MS) for the separation, identification and quantification of main volatile compounds. The HS-SPME shows several advantages over other analytical techniques since it is easy to use, is not consuming and is free of organic solvents [13]. However, several critical factors should be considered in order to improve the efficiency of HS-SPME analysis, such as the selection of the appropriate fiber coating, extraction temperature, sample weight, time of extraction, among others [14]. For this purpose, a response surface methodology is proposed as a novel strategy for optimization of the most relevant HS-SPME operating factors with a reduced number of experimental runs, which has not been previously reported for celery samples [8,9,15,16]. Up to now, the identification and quantification of the main volatile compounds of different celery cultivars and parts of the plants, such as leaves [8,9] and the stalk [17], have been reported in the literature. However, none of these studies have applied optimization of the extraction of the HS-SPME procedure by using the experimental design methodology. Regarding the extraction temperature, the reported values of this parameter in studies carried out in celery samples are between 20–60 °C, while the most suitable extraction time is between 30 and 50 min and the sample weight is around 0.5 to 4.0 g, depending on the study.

Recently, the celery plant has been exploited as a natural food preservative due to its antioxidant properties [3]. In particular, the secondary metabolites of phenolic acids and flavonoids have been pointed out due to their strong antioxidant properties [17]. Several studies underlined the in vitro antioxidant activity of different celery cultivars [18,19,20,21] and parts of the plant, such as the root [5,19], leaves and petioles [4]. To date, there is no unique relevant method to evaluate the in vitro antioxidant activity in food matrices. The antioxidant capacity by the DPPH^•^ (1,1-diphenyl-2-picrylhydrazyl) method has been reported for celery roots showing a scavenging capacity ranging from 65–70% for extracts diluted by ethanol to concentrations ranging from 0.2 to 6 mg mL^−1^ [22]. In celery stalks samples, the DPPH method showed a scavenging capacity that ranged from 23–39% when 5 g of the sample was extracted with 100 mL of 95% ethanol [4]. The ABTS^•^ (2,20-azinobis(3-ethylbenzthiazoline-6-sulfonate)) and Iron Reducing Power (FRAP) methods have also been used for celery samples obtaining values near 200 and 252 μmol Trolox g^−1^ dry weight, respectively [21]. Eleven cultivars of celery showed a DPPH and ABTS results in the range of 86–106 and 82–114 μM Trolox per 100 g fresh weight, respectively [22]. In particular, phenolic substances are known to be mainly responsible for the antioxidant activity of celery plants, and thereby for their physiological functionalities, for instance, as an anti-inflammatory, lowering cholesterol levels and anticancer activities [22]. Thus, the total phenolic content (TPC) has been reported for celery roots ranging from 265.44–368.51 μmol of chlorogenic acid per g of dry extract [20], whereas values from 1.7–5.0 mg gallic acid equivalent per 100 g (dry weight) have been reported for different celery cultivars [21,22].

The aim of the present work was the study of the potential applicability of industrial celery (*Apium graveolens*) by-products as aromatic and antioxidant sources. Thus, the volatile component profiles in the stalk and root samples were analyzed by HS-SPME–GC–MS, and their total phenolic content (TPC) and antioxidant activities determined by two different methods (ABTS and FRAP).

## 2. Materials and Methods

### 2.1. Reagents

Sigma-Aldrich Inc. (St. Louis, MO, USA) was used to acquire limonene, γ-terpinene, 2-methyl-1-pentanol, 2,4,6-tris(2-pyridyl)-s-triazine (TPTZ), 2,2-azinobis (3-ethylbenzothiazoline-6-sulfonic acid) diammonium salt (ABTS), Folin and Ciocalteu’s phenol reagent (2 N), gallic acid monohydrate and Trolox ((±)-6-hydroxy-2,5,7,8-tetramethylchromane-2-carboxylic acid). N-hexane (99%, GC grade), potassium persulfate, methanol (HPLC grade), sodium chloride, ferric chloride, glacial acetic acid and sodium carbonate were obtained from Panreac (Barcelona, Spain).

### 2.2. Celery By-Products Preparation

**Figure 1:**
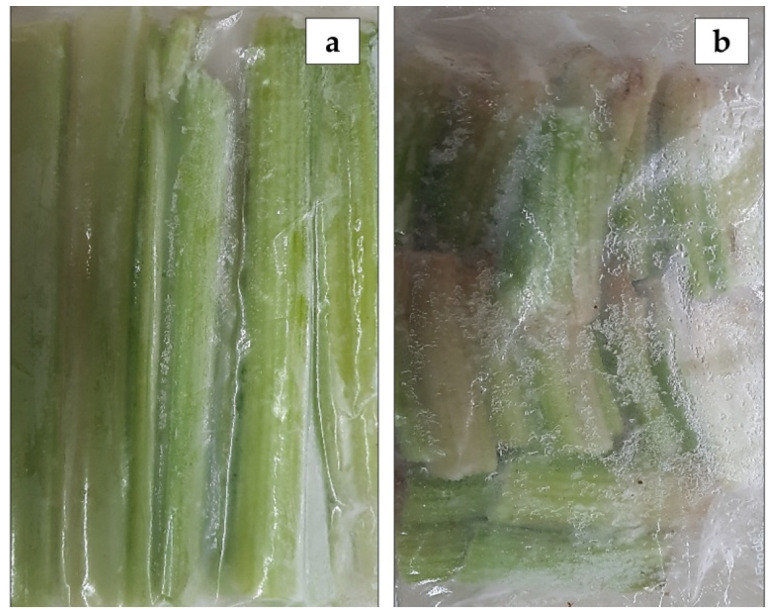
Celery by-products used in this study: (**a**) stalk; (**b**) root.

Two different by-products of celery samples, the stalk and the root, provided from Anecoop S. Coop (Murcia, Spain) were included in the study. All samples were grown in the eastern Mediterranean area of Spain, in the Valencian community with mean temperatures between 15 and 25 °C. They were all collected in February 2021. No postharvest process was practiced after the harvesting procedure. To prevent the loss of aromatic components and antioxidant capacity, when they arrived at the laboratory, the samples were immediately washed with cold distilled water and dried at room temperature, then vacuum-packed and frozen at −21 °C until they were subject to further analysis in the laboratory (Figure 1). Three batches of each by-product (M_1_, M_2_ and M_3_ of stalks and M_4_, M_5_ and M_6_ of roots) were analyzed in triplicate. Prior to the analysis, the samples were firstly crushed with a domestic blender for 20 s in order to reduce their size and to obtain homogenized samples.

The crushed samples were used directly for the analysis by HS-SPME–GC–MS, while the extraction of the antioxidant compounds was required for the determination of the TPC and the evaluation of antioxidant capacity. For the extraction of the antioxidant compounds, the procedure was carried out in triplicate according to the following procedure: 1.0 ± 0.1 g of grounded celery by-products were extracted with 4 mL of methanol/deionised water/HCl mixture (1%) (70:29:1) during 1 min in a vortex and let to stand for 5 min. Then, the sample was centrifuged at 5000 rpm for 10 min at room temperature and the supernatant was collected to a new tube with a Pasteur pipette. The extraction process was repeated twice by using the solid sample. The combined supernatants and grounded samples were stored at −18 °C until analysis.

### 2.3. Response Surface Methodology and HS-SPME Extraction Procedure

The HS-SPME extraction conditions were optimized using a Box–Behnken design (BBD). The optimization of the following three variables was carried out at three levels: the sample weight (g), the extraction temperature (°C) and the extraction time (time). Seventeen experiments, including five central points, were generated by a BBD model and executed in a randomized order. The factor levels and the experimental domain are shown in Table 1. The total area of the following identified volatile compounds (VOCs) was used as the response: six monoterpenes (limonene; camphene; α-pinene; β-pinene; β-cymene; γ-terpinene), three sesquiterpenes (caryophyllene; selinene; humulene), one alcohol (epiglobulol), one furan (2-pentylfuran) and one aromatic hydrocarbon (pentylbenzene). All of them have been reported in the literature as contributing significantly to the aroma of celery [6,8].

Analysis of samples was performed using a divinylbenzene/carboxen/polydimethylsiloxane (DVB/CAR/PDMS) fiber (Supelco, Bellefonte, PA, USA). The procedure was carried out using the auto-sampler. This fiber has been successfully used to extract volatile compounds from celery roots and aerial parts [8] and other vegetable matrices, such as capsicum [23], carrot [24,25] and onion [26,27]—all of them with a high content of fiber in their composition. To verify for possible carry-over effects, a blank test was performed before each analysis. A required amount of sample, 5 μL of the internal standard (2-methyl-1-pentanol; 560 mg Kg^−1^) and 2 mL of distilled water were added in a 20 mL hermetically sealed vial. The headspace of the sample was exposed to the fiber and maintained in contact for the required time and extraction temperature. All volatile extractions were made under constant stirring at 600 rpm. Subsequently, after extraction, the fiber was immediately desorbed for 10 min at 250 °C into the GC injector port.

### 2.4. GC–MS Conditions

Fiber desorption was performed in the splitless mode using an Agilent 7890B GC coupled to a mass spectrometer Agilent 5977B (Agilent Technologies Inc., Palo Alto, CA, USA) device. A Gerstel Multipurpose Sampler (MPS) robot (Agilent Technologies Inc., Palo Alto, CA, USA) was used as the sample introduction system to the chromatograph. The chromatographic column used was a DB-624 capillary column (30 m × 0.25 mm i.d., df = 1.40 μm). The sample was subjected to the following temperature program: initial temperature of 40 °C, maintained for 2 min and then an increase in temperature at a rate of 5 °C min^−1^ up to 250 °C, where it was maintained for 10 min. The carrier gas was purified helium at 1.0 mL min^−1^. MS data were recorded between 30–550 *m*/*z*, with an electron energy of 70 eV. The temperature values of the ion source and the transfer line were 230 and 150 °C, respectively.

The volatiles identification was achieved by comparison of the mass spectra of each compound with the NIST data library (v.2.0d software; Washington, DC, USA, 2005). Only the volatile compounds that had 80% similarity or higher were identified. The relative quantities of the volatile compounds are expressed as percent peak areas relative to the total peak area of identified compounds. In this study, two main celery volatile markers were selected for quantification in triplicate using as standards limonene and γ-terpinene. Both compounds were the most abundant compounds in HS-SPME optimal conditions for all studied samples. The selected compounds were quantified using calibration curves at six concentration levels dissolved in deionized water.

### 2.5. Antioxidant Activity

In this study we propose the use of an ABTS radicals-scavenging ability assay, as well as an iron-reducing capacity (FRAP) methodology to give a comprehensive prediction of antioxidant efficacy, using a spectrophotometer (Biomate-3, Thermospectronic, Mobile, AL, USA). All determinations were performed in triplicate.

#### 2.5.1. ABTS

The ABTS radical cations were prepared according to Yao et al. with some modifications [19]. 300 µL volumes of the extracts were vortexed with 3.0 mL of the ABTS solution. After incubation for 30 min at 25 ± 2 °C, the absorbance was measured at 734 nm. Trolox ethanolic solutions were used as standard. The results were expressed in μM Trolox per 100 g fresh weight (FW).

#### 2.5.2. FRAP

The antioxidant capacity of celery by-products was also assayed based on a ferric-reducing antioxidant power (FRAP) method as described by Benzie and Strain [28]. To obtain the FRAP reactive, 2.5 mL of 20 mM FeCl_3_, and 2.5 mL of 10 mM TPTZ were mixed with 25 mL of 300 mM sodium acetate buffer (pH 3.6, adjusted with acetic acid). For this analysis, a 200 μL of ethanol extract was mixed with 3.0 mL of FRAP reagent in a 5 mL polypropilene tube. After incubation at 20 °C for 30 min, absorbance was measured at 593 nm wavelength against a blank. Trolox dissolved in ethanol in appropriate dilution was used as a standard. The results were expressed in μM Trolox per 100 g fresh weight (FW).

### 2.6. Total Phenolic Content

Folin–Ciocalteu reagent was used to determine in triplicate the total phenol level of samples. The TPC assay was performed according to Al-Moubaraki et al. [11] with some modifications. The Folin-Chiocalteu’s phenol reagent (100 µL) was mixed with the methanolic extract (500 µL), distilled water (200 µL) and a 7% sodium carbonate aqueous solution (500 µL). After incubation for 90 min at room temperature protected from light, the absorbance was measured in a spectrophotometer (Biomate-3, Thermospectronic, Mobile, AL, USA) at 760 nm using the extractant as the blank. The results were expressed as mg gallic acid equivalents (GAE) per 100 g of fresh weight (FW).

### 2.7. Statistical Analysis

StatGraphics Centurion XV software (Statistical Graphics Corporation, Rockville, MD, USA) was used to maximize the response obtained from the fitted model. The adequacy of the model was determined by evaluating the lack of fit, the coefficient of determination (R^2^) and F-test obtained from the analysis of variance (ANOVA). The statistical significance of model parameters was determined at a 5% probability level (α = 0.05). Additional confirmation experiments were conducted (in triplicate) to verify the model validation. SPSS commercial software, ver. 15.0 (IBM, Chicago, IL, USA) was used for ANOVA analysis, the Pearson correlation coefficient (R) and *p*-value were used to show correlations and their significances. A Tukey test was assessed at a *p* ≤ 0.05 significance level to obtain differences between values.

## 3. Results and Discussion

### 3.1. Optimization of HS-SPME Procedure

The parameters considered during HS-SPME optimization were sample weight (0.5, 1.75, 3.0 g), extraction temperature (30, 50, 70 °C) and extraction time (10, 35 and 60 min). Table 1 shows the actual results of volatile compounds extraction (sum of areas) as a response to the different extraction conditions performed in the BBD. These parameters were selected because they may impact the HS-SPME extraction efficiency due to their strong influence on vapor pressure and the equilibrium between the concentration of volatile compounds in the headspace and the sample solution [19]. This range of studied variables was selected on the basis of results obtained in preliminary experiments and previous studies reported in the literature [20].

With regard to sample weight, it was found that 2 mL of distilled water was the more effective volume to be used, with a maximum quantity of 3.0 g of samples without the formation of sample aggregates in the vial during HS-SPME extraction. Concerning the extraction temperature, higher temperatures than 70 °C were not used to avoid the possibility of the degradation of some analytes [7,21]. Finally, it has been reported that the occupation of fiber sites by analytes is enhanced with longer extraction times. However, the pre-concentration efficiency is not affected when all sites are completed when the extraction time is prolonged and can even sometimes cause desorption [4,22].

**Figure 2:**
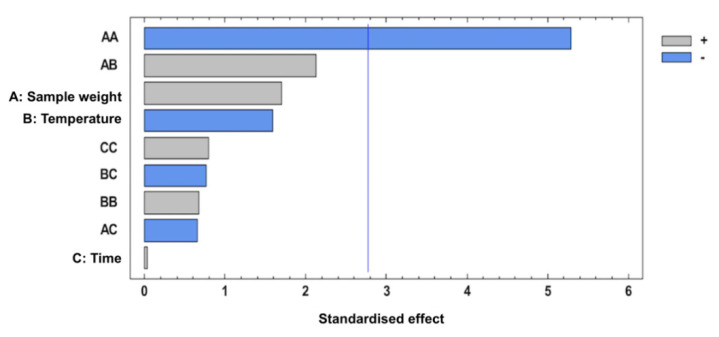
Pareto chart of the standardized effects of a BBD for the total obtained response area.

A Pareto chart (Figure 2) illustrates that only the quadratic effect of sample weight (AA) had a statistically significant influence on the sum of response areas, whereas the rest of the variables and interactions were not statistically significant. In this sense, the Pareto chart reveals that an increase of sample weight has a negative effect on the absorption of volatile compounds present in celery on to the fiber that could be related with the saturation phenomenon of the HS and, subsequently, the fiber coating [23,24,29]. Regarding the positive influence of the quadratic effect of BB, it has been previously reported that a low temperature could be insufficient for the volatilization of organic compounds [29].

**Figure 3:**
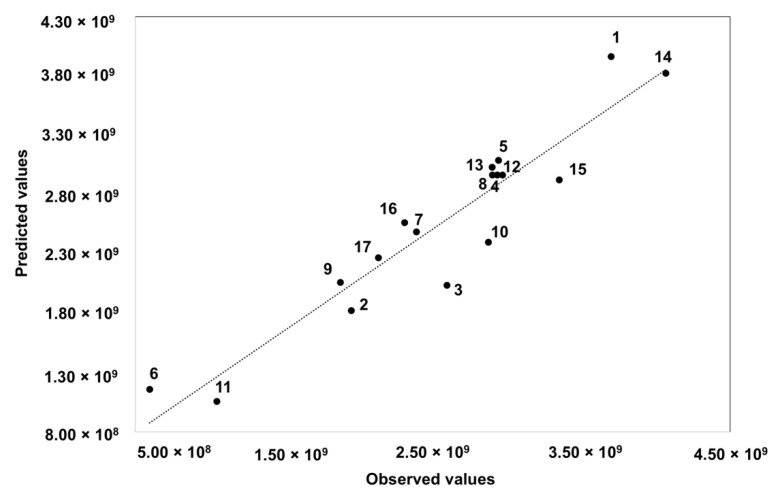
Predicted versus observed values of a BBD design.

**Figure 4:**
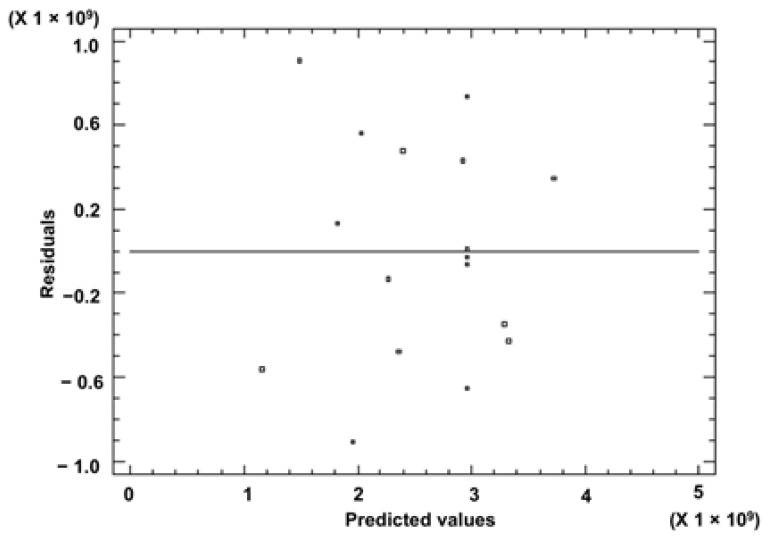
Residual versus predicted values of a BBD design.

The determination coefficient (R^2^) of the quadratic regression model was 0.6446, which shows that the sum of areas can be explained and predicted by the generated model. A value of 0.0867 (*p* > 0.05) was obtained for the ‘Lack of Fit’ test suggesting that the model was reliable in predicting the response and that it can predict the variations within the stalk. Therefore, the model explained the response adequately. Figure 3 shows the plot of the predicted versus the observed values that confirms the good fitting ability, whereas Figure 4 showed the residuals behavior with no significant trend.

The regression equation of response (total area), depending on the sample weight (A), extraction temperature (B) and extraction time (C), and the response (total area), determined on the basis of the second-order polynomial equation—Equation (1)—was:

Response = 2.85 × 10^9^ + 2.28 × 10^9^ × A − 8.16 × 10^7^ × B + 1.44 × 10^7^ × C − 7.71× 10^8^ × A^2^ + 1.56× 10^7^ × A × B − 5.19 × 10^6^ × A × C + 570, 835 × B^2^ − 379, 394 × B × C + 197, 762 × C^2^ (1)

**Figure 5:**
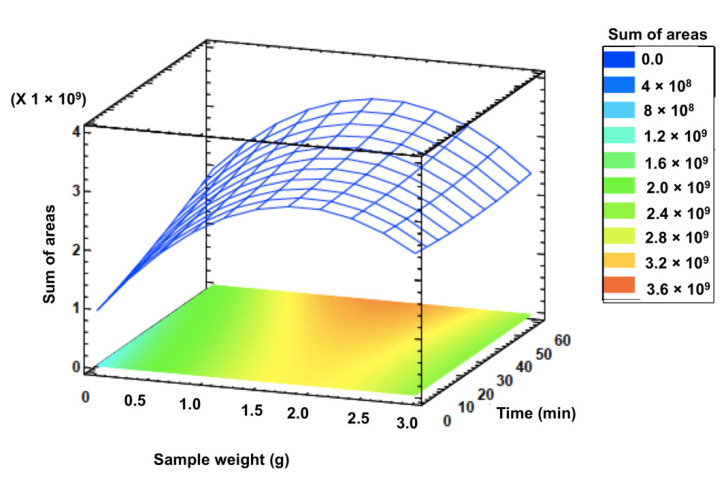
Surface response and contour plot for the interaction between sample weight and time when temperature is fixed at the optimum level.

The optimal extraction conditions obtained in the present study were 1.6 g of celery sample at 30 °C for 60 min. Under these conditions, the response value predicted by the model was 3.74 × 10^9^. To verify the model, three experiments were performed under optimal conditions with experimental values of 4.38 × 10^9^ ± 2.21 × 10^7^, which did not differ significantly from the predicted values. Figure 5 reports the response surface plot of signal intensity as a function of sample weight and time when the extraction temperature is at the optimum level (30 °C). In this study, a lower extraction temperature, and a longer time of extraction is recommended to prevent the formation of artifacts [25], this being in accordance with the Pareto chart (Figure 2) in which a negative effect was observed for BC. Although the extraction time was not statistically significant in Figure 2, the positive effect of C and its quadratic effect CC is noticeable, suggesting that extraction increases with time. In this sense, the mass transfer of the analytes between the three phases in the HS-SPME technique is affected by the time extraction, mainly determined by the partition coefficient of the analyte between the fiber coating and sample matrix [26]. This is in accordance with previous studies in which the extraction efficiency of the HS-SPME step increase with extraction time [30]. Concerning the extraction temperature, a decrease in volatiles extraction could be explained by their degradation, as has been previously reported [27,31]. Finally, to increase the HS volume without affecting the extraction efficiency of the process, the reduction of the sample volume in a constant vial size is highly recommended [32,33,34]. In this work, a medium sample weight of 1.6 g was obtained as the optimum level.

### 3.2. Volatile Compounds

By optimizing the HS-SPME–GC–MS procedure, 26 volatile compounds were identified, and their relative abundance was determined in the stalk and root celery wastes. Table 2 shows the volatile compounds and the retention times used for the identification of the compounds, together with the main sensory descriptors of each of the volatiles.

In this work, celery wastes showed a variety of compounds that contribute to its odour and aroma. Nine chemical families were successfully identified in stalk and root samples: nine monoterpenes (α-pinene, camphene, sabinene, β-pinene, limonene, β-cymene, γ-terpinene, terpinolene and dihydrocarvone), six sesquiterpenes (cyclosativene, α-copaene, caryophyllene, humulene, β-selinene and δ-cadinene), three alcohols (2,3-butanediol, (Z)-carveol and globulol), two ketones (β-ionone and 3-hydroxybutanone), two aromatic hydrocarbons (m-xylene and pentylbenzene), one furan (2-pentylfuran), one alkene (6-Butyl-1,4-cycloheptadiene), one phtalide (3-butylphthalide) and one phenol (4-heptylphenol). Among identified volatile compounds, several common compounds were previously detected in *Apium graveolens L.* leaves by HS-SPME–GC–MS (α-pinene, camphene, β-pinene, limonene, caryophyllene, humulene and 3-butylphtalide, β-cymene, γ-terpinene, terpinolene, dihydrocarvone, (Z)-carveol, α-copaene, humulene, β-selinene and δ-cadinene) [8,9] and in the essential oil of the aerial part of celery (sabinene, pentylbenzene and globulol) [14,35]. As a result, woody, herbal and citrus odor descriptors could be associated with these compounds. The rest of the volatile compounds identified in Table 2 (3-hydroxybutanone, 2,3-butanediol, m-xylene, 2-pentylfuran, 6-butyl-1,4-cycloheptadiene, cyclosativene, β-ionone and 4-heptylphenol) have been reported in other different vegetable matrices, such as blueberries [36], grapes [37], the root and leaf of *Rumex Crispus I.* [38], onions [26], capsicum [23] and greengage fermentation waste [39].

**Figure 6:**
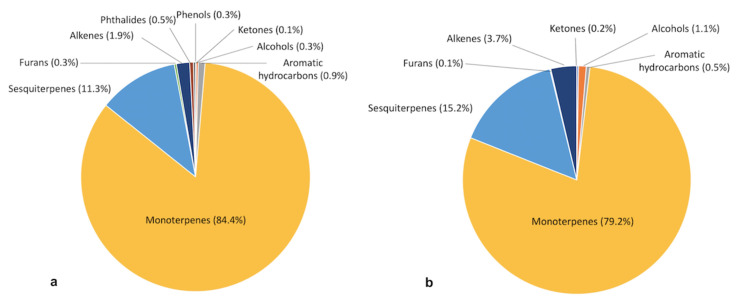
Distribution (%) of chemical groups of volatile compounds in celery stalk (**a**) and root (**b**).

To make the discussion of this section easier, the average distribution (%) of chemical groups of volatile compounds in the celery stalk and root are summarized in Figure 6. The volatile composition of stalk waste (Figure 6a) was dominated by monoterpenes (84.4%) and sesquiterpenes accounted for about 11.3%, followed by alkenes (1.9%) and other minor compounds, such as the non-terpenoid aromatic hydrocarbon, m-xylene (0.9%), phthalides (0.5%), furans, alcohols and phenols (0.3%, respectively) and ketones (0.1%). It can be observed that the volatile profile differed from the root waste (Figure 6b) in which the monoterpenes (79.2%) and sesquiterpenes (15.2%) are still dominant but with different concentrations, followed by alkenes (3.7%) and other minor compounds, such as alcohols (1.1%), aromatic hydrocarbons (0.5%), ketones (0.2%) and furans (0.1%). In the root sample, no phthalides and phenols were found. This agrees with a previous study where the factors that influence the aroma profile of celery (*Apium graveolens*) are reviewed [6], indicating that the aroma compounds present in celery are different depending on the part of the plant used.

The main contributors to the characteristic citrus and herbal aroma of celery are terpenoids [6,8,9], sesquiterpenes made up of three isoprene units (C_15_H_24_) and monoterpenes with two isoprene units (C_10_H_16_), which agrees with the results obtained in the present study. Table 3 shows the variation in percentage composition between the main volatile compounds of the studied samples. In this work, certain compounds showed similar behavior in all samples. In this sense, stalk and root samples were rich in limonene and γ-terpinene, which were the predominant identified volatiles, this being in accordance with the literature since an abundance of monoterpenes have been identified in celery [5,8]. In particular, limonene is the most abundant terpene contributing to the citrus, lemon and fresh aromas of celery [16,41,42]. It seems that stalk samples showed a higher content of limonene in contrast to the roots, whereas the content of γ-terpinene varied between samples. Similar behavior observed for the γ-terpinene is represented in Table 3 for the rest of the studied compounds, suggesting that multiple factors play a role in celery flavor, including geographical location, the particular cultivar and the material of the plant that is used [9]. This having been said, it seems that the stalk and root samples were rich in other terpenes including β-cymene, followed by caryophyllene, β-pinene and, lastly, β-selinene.

Finally, other minor compounds were detected in the studied samples, such as ketones, alcohols, phthalides and others. In plants, alcohols and ketones are formed from saturated and unsaturated fatty acids, such as linolenic acid by three different pathways: α-oxidation, β-oxidation and the lipoxygenase via [6,43]. Phthalides in plants are derived from 1(3H)-isobenzofuranone consisting of one benzene ring bonded with a γ-lactone between carbon atoms [44,45,46,47]. In particular, the 3-butylphthalide is considered to contribute to the fresh aroma of celery [6].

### 3.3. Quantification of Limonene and γ-Terpinene Compounds

**Figure 7:**
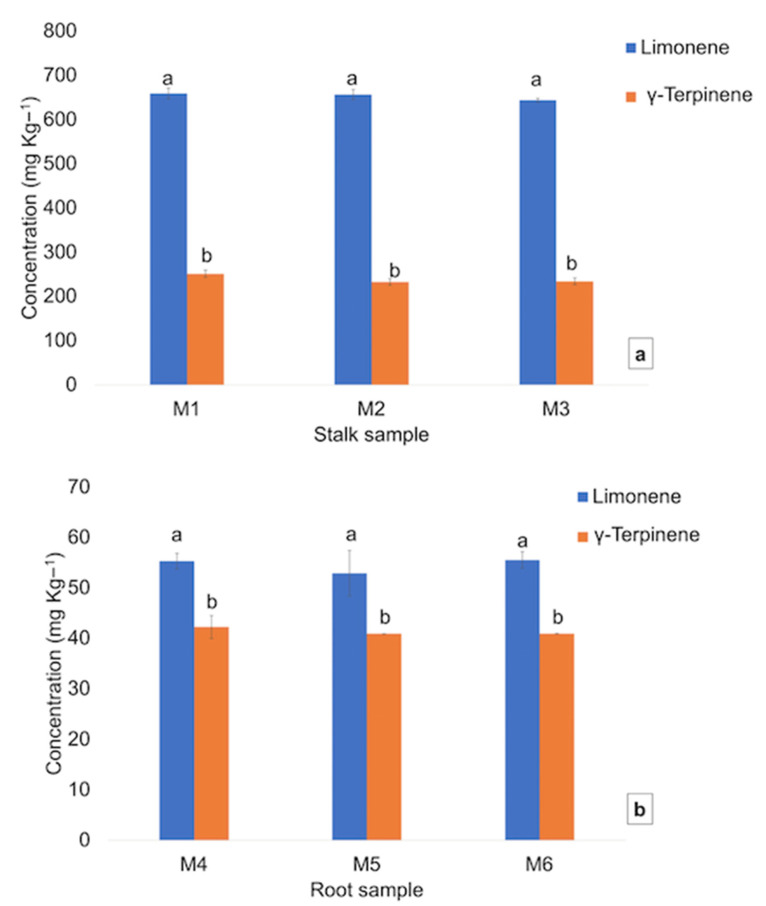
Volatile concentration (mg Kg^−1^) of limonene and γ-terpinene in celery stalk (**a**) and root (**b**) samples. Values (*n* = 3 ± SD) followed by the same letter, within the same volatile compound, were not significantly different (*p* < 0.05), according to Tukey’s least significant difference test.

The quantification of the two main volatile compounds in the studied samples showed R^2^ values of 0.9794 for limonene and 0.9852 for γ-terpinene. Figure 7 shows the main differences observed for stalk (M_1_, M_2_ and M_3_) and root samples (M_4_, M_5_ and M_6_) in relation to both quantified components. In general, all samples showed a higher content of limonene than γ-terpinene, in accordance with the results shown in Table 3. However, significant differences in limonene and γ-terpinene content were observed between the stalk and root wastes.

Regarding the celery stalk samples (Figure 7a), no statistically significant differences were observed for M_1_, M_2_ and M_3_ regarding limonene, with values of 659.0 ± 12.3, 656.6 ± 12.3 and 644.0 ± 4.2 mg Kg^−1^, respectively, and γ-terpinene with values of 251.1 ± 8.3, 245.7 ± 7.4 and 243.9 ± 7.5 mg Kg^−1^, respectively. Concerning the root celery samples (Figure 7b), no statistically significant differences were observed for M_4_, M_5_ and M_6_ regarding limonene (55.3 ± 1.5, 52.9 ± 4.5 and 55.6 ± 1.6 mg Kg^−1^, respectively) and γ-terpinene (42.3 ± 2.3, 40.9 ± 0.5 and 41.0 ± 0.9 mg Kg^−1^, respectively). However, it is noticeable that stalk samples had a higher content in both volatiles in contrast to the root samples. From this study, it may be possible to conclude that the overall differences in volatile composition for *Apium graveolens* wastes could reside in the part of the plant taken in the study being in accordance with the reported literature [3,4,6]. In plants, it has been identified that fatty acids, carbohydrates and amino acids are the original precursors for the biosynthetic pathways that lead to volatile synthesis [38,43]. Terpenoids are enzymatically synthesized from acetyl CoA and pyruvate provided by the carbohydrate pools in plastids and the cytoplasm. Consequently, it could be suggested that the composition of these tissues differ.

### 3.4. Antioxidant Activity

In this study the use of ABTS and FRAP methodology to give a comprehensive prediction of antioxidant efficacy of celery by-products is proposed. Both methods are expressed as μM Trolox per 100 g fresh weight (FW) and the obtained results in samples of celery roots and stalks are shown in Table 4.

The FRAP method in stalk celery waste samples showed values from 328.3 to 368.7 μM Trolox 100 g^−1^ FW, whereas, in the root samples, values between 172.4 and 389.9 μM Trolox 100 g^−1^ FW were obtained. In general, no statistically significant differences were obtained between samples (*p* < 0.05), with the exception of sample M_4_. The content differences between the samples could very well be due to environmental differences, including locations, postharvest treatments, pest exposure and the choice of parts to be tested [9]. However, in general, it could be concluded that both residues, stalks and roots showed a similar average range. A similar trend was observed when antioxidant activity was measured by the ABTS method, since stalk samples show values from 63.1 to 232.2 μM Trolox 100 g^−1^ FW, whereas, in the root samples, values between 85.8 and 196.7 μM Trolox 100 g^−1^ FW were obtained. These results were similar to those reported for eleven cultivars of celery from China with an average value ranging from 81.9 to 114.38 μM TE 100 g^−1^ for the samples [22] and two celery cultivars (Shengjie celery (*A. graveolens* L.) and Tropica (*C. japonica* Hassk.)) with an average value ranging from 87.4 to 584.9 μM TE 100 g^−1^ of FW [19]. Generally, from these results, it was concluded that the studied celery residues have antioxidant capacities.

### 3.5. Total Phenolic Content

Table 4 shows the results obtained by the Folin–Ciocalteu method. The TPC average range values of celery stalks were 16.3–49.0 mg GAE 100 g^−1^ FW, whereas, for the root samples, values between 14.6 and 33.2 mg GAE 100 g^−1^ FW were achieved. These results were similar to those reported for two cultivars of celery from China with an average value ranging from 26.0 to 34.6 mg GAE 100 g^−1^ FW [19]. From these results it could be concluded that it seems the stalk showed higher TPC values in contrast to the root. This fact could be explained by phenolic components which can vary considerably even among different tissues of the same cultivar [4]. In this sense, phenolic acids and flavonoids have been reported as typical phenolic compounds that possess antioxidant activity [48]. In fact, *p*-coumaric acid was the predominant compound reported in the literature in celery stalk extracts, tailed by chlorogenic acid, caffeic acid, luteolin, quercetin, gallic acid, ferulic acid, rutin, syringic acid and vanillic acid [18]. Concerning celery roots, the main phenolic compounds previously reported were catechin, 3,4-dihydroxybenzoic acid, 1,2-dihydroxybenzene and gallic acid [18,20].

### 3.6. Correlation of Total Phenolic Content with Antioxidant Activity

A linear correlation (Pearson’s correlation square coefficient) between the TPC and their antioxidant capacity has been previously reported in the literature in numerous fruits and vegetables, such as different celery cultivars [19,22,49]. A significant correlation between TPC and the FRAP assay (R = 0.645; *p* < 0.01) was obtained in the present work. TPC is also highly correlated with the ABTS assay (R = 0.905; *p* < 0.01), indicating that total phenolic acids contributed to the antioxidant capacity of samples. In addition, ABTS and FRAP methods demonstrated an adequate but not very high correlation coefficient (R = 0.635; *p* < 0.01). This fact may be due to chemical dissimilarity between the two assays, since the ABTS method is based on the electron transfer reaction whereas the FRAP assay is based on the reduction of the Fe^3+^–TPTZ complex to its colored ferrous form (Fe^2+^–TPTZ) in the presence of antioxidant compounds [50]. This could explain the observed differences between the results in Table 4, since it is shown that in general the FRAP method underlined a higher average value of μM TE 100 g^−1^ in samples than ABTS.

## 4. Conclusions

The HS-SPME extraction technique combined with GC–MS has been shown to be a rapid, automated and solvent-free method for the analysis of the volatility profile in celery by-products. In this work, the volatile compounds’ extraction conditions were optimized by employing a Box–Behnken design and the optimal extraction conditions were achieved with 1.6 g of celery sample at 30 °C for 60 min. Twenty-six volatile compounds were isolated and identified in the stalk and root celery wastes under optimal conditions. Differences regarding the volatility profile of stalk and root were observed since stalk waste was dominated by monoterpenes and sesquiterpenes, followed by alkenes and other minor compounds (non-terpenoid aromatic hydrocarbon, phthalides, furans, alcohols, phenols and ketones) whereas the root waste was still dominated by monoterpenes and sesquiterpenes but with different concentrations, followed by alkenes and other minor compounds (alcohols, aromatic hydrocarbon, ketones and furans with no phthalides and phenols present). Two main monoterpenes were predominant in stalk and root samples: limonene and γ-terpinene. Their quantification underlined the potential of the stalk waste as a rich source of both volatile compounds in contrast to the root, which could be further studied for its potential as a citrus aromatic additive with industrial applications.

Antioxidant activities and the TPC of the studied samples underlined the studied celery wastes (stalk and root) as good sources of free radical scavengers. These results provided a scientific basis for assessing the industrial applications of celery by-products, e.g., as additives for food packaging or as functional food ingredients. Additionally, its use as a natural preservative could reduce the need for applying artificial preservatives, additives and antioxidants in food, contributing to the well-known circular economy.

## Figures and Tables

**Table 1:** Combinations of experiment conditions of the Box–Behnken design (BBD) and the measured sum of areas of common volatile compounds present in celery by-products.

Run	Sample Weight (g)	Temperature (°C)	Time (Min)	Sum of Areas
**1**	1.75	50	35	3.70 × 10^9^
**2**	1.75	50	35	2.90 × 10^9^
**3**	3.0	50	60	1.05 × 10^9^
**4**	1.75	70	10	2.94 × 10^9^
**5**	1.75	30	10	2.90 × 10^9^
**6**	0.5	50	10	2.39 × 10^9^
**7**	1.75	70	60	3.35 × 10^9^
**8**	3.0	50	10	2.14 × 10^9^
**9**	0.5	30	35	1.88 × 10^9^
**10**	3.0	70	35	2.88 × 10^9^
**11**	1.75	30	60	4.07 × 10^9^
**12**	1.75	50	35	2.94 × 10^9^
**13**	0.5	70	35	5.97 × 10^8^
**14**	1.75	50	35	2.97 × 10^9^
**15**	3.0	30	35	2.60 × 10^9^
**16**	1.75	50	35	2.31 × 10^9^
**17**	0.5	50	60	1.95 × 10^9^

**Table 2:** Volatile compounds identification, retention times and sensory descriptors obtained by headspace solid phase microextraction–gas chromatography–mass spectrometry (HS-SPME–GC–MS) in stalk and root celery wastes.

Compound	Retention Time (Min)	Chemical Group	Odour Descriptor ^a^
3-hydroxybutanone	10.08	Methyl ketone	Buttery, fatty
2,3-butanediol	14.00	Alcohol	Creamy
M-xylene	14.31	Aromatic hydrocarbon	Plastic
α-pinene	16.16	Monoterpene	Herbal
Camphene	16.85	Monoterpene	Woody
Sabinene	17.77	Monoterpene	Woody
β-pinene	17.89	Monoterpene	Terpenic
2-pentylfuran	18.40	Furan	Fruity, green, earthy
Limonene	19.74	Monoterpene	Lemon, orange, citrus
β-cymene	19.82	Monoterpene	Cumin, lemon
γ-terpinene	20.60	Monoterpene	Sweet, citrus
Terpinolene	22.99	Monoterpene	Herbal
6-butyl-1,4-cycloheptadiene	23.92	Alkene	Not identified
Pentylbenzene	24.12	Aromatic hydrocarbon	Not identified
Dihydrocarvone	26.80	Monoterpene	Herbal, minty
(Z)-carveol	27.07	Alcohol	Minty
Cyclosativene	30.33	Sesquiterpene	Not identified
α-copaene	30.63	Sesquiterpene	Woody, spicy, honey
Caryophyllene	32.01	Sesquiterpene	Sweet, woody, spice
Humulene	32.94	Sesquiterpene	Woody
β-selinene	33.74	Sesquiterpene	Herbal
δ-cadinene	34.21	Sesquiterpene	Herbal
β-ionone	34.48	Ketone	Floral
Globulol	34.89	Alcohol	Floral, rose
3-butylphthalide	39.81	Phthalide	Celery, herbal
4-heptylphenol	41.76	Phenol	Not identified

^a^ Odor description retrieved 25 September 2021 from http://www.thegoodscentscompany.com, last access 15 September 2021 [40].

**Table 3:** Relative abundance (%) of volatile compounds in stalk (M_1_, M_2_ and M_3_) and root (M_4_, M_5_ and M_6_) celery wastes.

Compound	M1	M2	M3	M4	M5	M6
Monoterpenes						
α-pinene	1.43 ± 0.03 ^a^	1.79 ± 0.03 ^b^	1.66 ± 0.02 ^c^	0.38 ± 0.01 ^d^	0.39 ± 0.01 ^d^	0.29 ± 0.02 ^e^
Camphene	0.26 ± 0.02 ^a^	0.28 ± 0.05 ^a^	0.27 ± 0.04 ^a^	0.10 ± 0.01 ^b^	0.05 ± 0.01 ^c^	0.03 ± 0.01 ^c^
Sabinene	0.06 ± 0.03 ^a^	0.18 ± 0.06 ^a^	0.10 ± 0.05 ^a^	1.76 ± 0.06 ^b^	1.34 ± 0.06 ^c^	0.54 ± 0.04 ^d^
β-pinene	3.43 ± 0.06 ^a^	1.74 ± 0.08 ^b^	2.11 ± 0.05 ^b^	6.71 ± 0.38 ^c^	5.16 ± 0.25 ^d^	5.18 ± 0.62 ^d^
Limonene	33.16 ± 2.46 ^a^	35.72 ± 1.51 ^a^	36.02 ± 0.48 ^a^	28.64 ± 0.74 ^b^	29.82 ± 1.20 ^b^	27.44 ± 0.70 ^b^
β -cymene	19.17 ± 1.93 ^a^	22.57 ± 0.74 ^b^	23.70 ± 0.54 ^b^	13.55 ± 0.55 ^c^	13.63 ± 0.55 ^c^	15.60 ± 0.69 ^c^
γ-terpinene	24.95 ± 0.89 ^a^	23.51 ± 0.91 ^a^	24.28 ± 0.76 ^a^	23.96 ± 1.07 ^a^	25.30 ± 0.54 ^ab^	26.59 ± 0.16 ^b^
Terpinolene	0.24 ± 0.05 ^a^	0.29 ± 0.07 ^a^	0.20 ± 0.09 ^a^	0.87 ± 0.07 ^b^	2.29 ± 0.10 ^c^	1.79 ± 0.16 ^d^
Dihydrocarvone	0.23 ± 0.02 ^a^	0.06 ± 0.01 ^b^	0.03 ± 0.02 ^b^	0.18 ± 0.03 ^c^	0.46 ± 0.04 ^d^	0.20 ± 0.02 ^ac^
Sesquiterpenes						
Cyclosativene	0.25 ± 0.50 ^a^	0.28 ± 0.05 ^b^	0.20 ± 0.02 ^c^	0.02 ± 0.01 ^d^	0.02 ± 0.01 ^d^	0.03 ± 0.01 ^d^
α-copaene	1.32 ± 0.10 ^a^	0.02 ± 0.01 ^b^	0.03 ± 0.01 ^b^	0.24 ± 0.01 ^c^	0.12 ± 0.01 ^b^	0.07 ± 0.01 ^b^
Caryophyllene	8.57 ± 0.22 ^ac^	7.13 ± 0.39 ^b^	7.59 ± 0.43 ^ab^	9.97 ± 0.60 ^c^	6.58 ± 0.62 ^bd^	5.24 ± 0.52 ^d^
Humulene	0.55 ± 0.06 ^ad^	0.07 ± 0.02 ^b^	0.64 ± 0.02 ^a^	2.84 ± 0.12 ^c^	0.55 ± 0.03 ^ad^	0.45 ± 0.02 ^d^
β-selinene	3.06 ± 0.18 ^a^	1.96 ± 0.08 ^b^	1.15 ± 0.06 ^b^	4.57 ± 0.32 ^c^	7.57 ± 0.64 ^d^	5.01 ± 0.31 ^c^
δ-cadinene	0.18 ± 0.03 ^a^	0.27 ± 0.05 ^a^	0.20 ± 0.04 ^a^	0.34 ± 0.01 ^b^	0.98 ± 0.07 ^c^	1.00 ± 0.03 ^c^
Ketones						
3-hydroxybutanone	0.03 ± 0.01 ^a^	0.01 ± 0.01 ^a^	0.01 ± 0.01 ^a^	0.16 ± 0.05 ^b^	0.22 ± 0.09 ^b^	0.19 ± 0.08 ^b^
β-ionone	0.12 ± 0.03 ^a^	0.12 ± 0.02 ^a^	0.11 ± 0.02 ^a^	0.10 ± 0.04 ^ab^	0.05 ± 0.03 ^b^	0.04 ± 0.02 ^b^
Alcohols						
2,3-butanediol	0.01 ± 0.01 ^a^	0.01 ± 0.01 ^a^	0.03 ± 0.01 ^a^	0.46 ± 0.04 ^b^	1.62 ± 0.51 ^c^	1.14 ± 0.15 ^c^
(Z)-carveol	0.15 ± 0.02 ^a^	0.15 ± 0.04 ^a^	0.28 ± 0.10 ^ab^	0.29 ± 0.01 ^b^	0.38 ± 0.03 ^c^	0.39 ± 0.02 ^c^
Globulol	0.15 ± 0.01 ^a^	0.15 ± 0.07 ^a^	0.29 ± 0.11 ^a^	nd	nd	nd
Others						
M-xylene	0.02 ± 0.01 ^a^	0.02 ± 0.01 ^a^	0.02 ± 0.02 ^a^	0.32 ± 0.20 ^b^	0.05 ± 0.01 ^c^	0.05 ± 0.02 ^c^
2-pentylfuran	0.31 ± 0.05 ^a^	0.30 ± 0.07 ^a^	0.28 ± 0.03 ^a^	0.21 ± 0.01 ^b^	0.09 ± 0.01 ^c^	0.10 ± 0.01 ^c^
6-butyl-1,4-cyclo Heptadiene	3.74 ± 0.09 ^a^	1.51 ± 0.09 ^b^	0.29 ± 0.12 ^c^	3.94 ± 0.04 ^a^	3.97 ± 0.17 ^a^	3.11 ± 0.55 ^a^
3-butylphthalide	0.49 ± 0.10 ^a^	0.58 ± 0.04 ^a^	0.54 ± 0.12 ^a^	nd	nd	nd
4-heptylphenol	0.73 ± 0.32 ^a^	0.07 ± 0.02 ^b^	0.06 ± 0.03 ^b^	nd	nd	nd
Pentylbenzene	1.50 ± 0.12 ^a^	1.23 ± 0.09 ^b^	0.06 ± 0.05 ^c^	0.28 ± 0.01 ^d^	0.37 ± 0.03 ^de^	0.52 ± 0.03 ^e^

nd: non-detected. Values (*n* = 3 ± SD) followed by the same letter (a, b, c, d, e), within the same row, were not significantly different (*p* < 0.05), according to Tukey’s least significant difference test.

**Table 4:** Antioxidant activity results measured by FRAP and ABTS methods and the TPC values of the celery wastes studied expressed as average quantities ± standard deviations (*n* = 3).

Sample	FRAP (μM Trolox 100 g^−1^ FW)	ABTS (μM Trolox 100 g^−1^ FW)	TPC (mg GAE 100 g^−1^ FW)
**M_1_**	328.3 ± 10.5 ^a^	63.1 ± 0.2 ^a^	16.3 ± 0.5 ^a^
**M_2_**	368.7 ± 39.9 ^a^	232.2 ± 25.0 ^b^	49.0 ± 5.4 ^b^
**M_3_**	355.7 ± 21.8 ^a^	144.4 ± 31.3 ^cd^	32.5 ± 3.7 ^c^
**M_4_**	172.4 ± 2.7 ^b^	85.8 ± 1.9 ^a^	14.6 ± 0.5 ^a^
**M_5_**	389.9 ± 24.8 ^a^	196.7 ± 25.1 ^bc^	33.2 ± 4.7 ^c^
**M_6_**	340.5 ± 28.5 ^a^	129.6 ± 11.5 ^d^	21.8 ± 2.9 ^a^

Different superscripts (a, b, c, d) for each parameter within the same column indicate statistically significant different values (*p* < 0.05).

## Data Availability

The data presented in this study are available on request from the corresponding author.
